# Tetra­aqua­diimidazole­nickel(II) naphthalene-1,5-disulfonate

**DOI:** 10.1107/S1600536807067761

**Published:** 2008-01-04

**Authors:** Ping Liu, Dong-Sheng Deng

**Affiliations:** a College of Chemistry and Chemical Engineering, Henan Institute of Science and Technology, Xinxiang, Henan 453003, People’s Republic of China; b College of Chemistry and Chemical Engineering, Luoyang Normal University, Luoyang 471022, People’s Republic of China

## Abstract

The triclinic unit cell of the title compound, [Ni(C_3_H_4_N_2_)_2_(H_2_O)_4_](C_10_H_6_O_6_S_2_), contains one centrosymmetric cation and one centrosymmetric anion. In the cation, the Ni^II^ ion is six-coordinated by two imidazole ligands [Ni—N = 2.0568 (14) Å] and four water mol­ecules [both independent Ni—O distances are 2.098 (1) Å] in a distorted octa­hedral geometry. Inter­molecular O—H⋯O and N—H⋯O hydrogen bonds form an extensive three-dimensional network, which consolidates the crystal packing.

## Related literature

For related literature, see: Côté & Shimizu (2003 ▶); Cai (2004 ▶); Cai *et al.* (2001 ▶); Chen *et al.* (2001 ▶, 2002 ▶); Lian *et al.* (2007 ▶); Liu *et al.* (2006 ▶); Zhou *et al.* (2004 ▶).
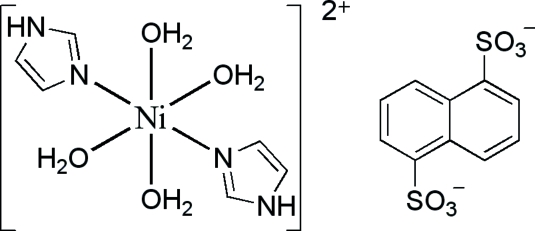


## Experimental

### 

#### Crystal data

[Ni(C_3_H_4_N_2_)_2_(H_2_O)_4_](C_10_H_6_O_6_S_2_)
*M* *_r_* = 553.21
Triclinic, 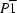

*a* = 8.285 (3) Å
*b* = 8.925 (3) Å
*c* = 9.088 (3) Å
α = 107.705 (5)°
β = 101.628 (5)°
γ = 111.967 (5)°
*V* = 554.6 (3) Å^3^
*Z* = 1
Mo *K*α radiation
μ = 1.12 mm^−1^
*T* = 273 (2) K
0.37 × 0.28 × 0.22 mm

#### Data collection

Bruker SMART 1K CCD diffractometer
Absorption correction: multi-scan (*SADABS*; Bruker, 2001 ▶) *T*_min_ = 0.681, *T*_max_ = 0.790
4834 measured reflections
2499 independent reflections
2262 reflections with *I* > 2σ(*I*)
*R*_int_ = 0.011

#### Refinement

*R*[*F* ^2^ > 2σ(*F* ^2^)] = 0.024
*wR*(*F* ^2^) = 0.067
*S* = 1.08
2499 reflections
167 parameters
6 restraints
H atoms treated by a mixture of independent and constrained refinement
Δρ_max_ = 0.35 e Å^−3^
Δρ_min_ = −0.25 e Å^−3^

### 

Data collection: *SMART* (Bruker, 1997 ▶); cell refinement: *SAINT* (Bruker, 2001 ▶); data reduction: *SAINT*; program(s) used to solve structure: *SHELXS97* (Sheldrick, 1997 ▶); program(s) used to refine structure: *SHELXL97* (Sheldrick, 1997 ▶); molecular graphics: *SHELXTL* (Bruker, 1997 ▶); software used to prepare material for publication: *SHELXTL*.

## Supplementary Material

Crystal structure: contains datablocks global, I. DOI: 10.1107/S1600536807067761/cv2373sup1.cif

Structure factors: contains datablocks I. DOI: 10.1107/S1600536807067761/cv2373Isup2.hkl

Additional supplementary materials:  crystallographic information; 3D view; checkCIF report

## Figures and Tables

**Table 1:** Hydrogen-bond geometry (Å, °)

*D*—H⋯*A*	*D*—H	H⋯*A*	*D*⋯*A*	*D*—H⋯*A*
O1*W*—H1*WA*⋯O3^i^	0.82 (2)	2.022 (15)	2.7507 (18)	148 (2)
O1*W*—H1*WB*⋯O1	0.82 (2)	2.018 (14)	2.788 (2)	156 (3)
O2*W*—H2*WA*⋯O1^ii^	0.81 (2)	1.962 (10)	2.7496 (19)	161 (2)
O2*W*—H2*WB*⋯O2^iii^	0.81 (2)	1.96 (2)	2.7979 (18)	173 (2)
N2—H7*A*⋯O3^iv^	0.86	2.19	2.981 (2)	154

Symmetry codes: (i) 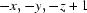; (ii) 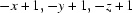; (iii) 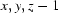; (iv) 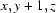.

**Fig. 1.**
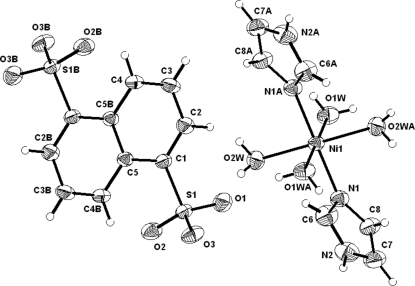
View of the title compound with the atomic numbering and 30% probability displacement ellipsoids [symmetry codes: (A) -x + 1, -y + 1, -z + 1; (B) -x + 1, -y, -z + 2].

## supplementary crystallographic information

### Comment

The weak coordination nature of SO_3_^-^ makes its coordination mode very flexible and sensitive to the chemical environment (Côté *et al.*, 2003). It is known that the coordination behavior of arenesulfonates with transition metals can be tailored in the presence of amino ligands (Lian *et al.*, 2007; Liu *et al.*, 2006; Zhou *et al.*, 2004; Chen *et al.*, 2001; Cai *et al.*, 2001; Chen *et al.*, 2002). Herewith we present the crystal structure of the title compound, [C_6_H_16_N_4_NiO_4_]^2+^.[C_10_H_6_O_6_S_2_]^2-^ (I) (Fig. 1).

The asymmetric unit of (I) contains a half of complex cation and a half of organic anion. Four water molecules coordinate to the nickel ion in *trans* position, respectively, and two imine nitrogen atoms from two imidazole ligands coordinate to nickel atom in *trans* position too. Thus, the nickel ion has a slightly distorted octahedral coordination geometry.

The title compound adopts the same hybrid organic-inorganic packing pattern as that reported earlier (Cai, 2004; Chen *et al.*, 2001; Cai *et al.*, 2001; Chen *et al.*, 2002). The intermolecular O—H···O and N—H···O hydrogen bonds (Table 1) form an extensive three-dimensional network, which consolidates the crystal packing.

### Experimental

Disodium naphthalene-1,5-disulfonate (0.33 g, 1 mmol) and imidazole (0.27 g, 4 mmol) were added to an aqueous solution of NiCl_2_6H_2_O (0.24 g, 1 mmol). The result solution was stirred at 60°C for four hours in a water bath. After filtration, a clear solution was set aside to crystallize. Platelike blue crystals were collected in 70% yield (base on Ni) after three days. Anal. Calcd for C_16_H_22_N_4_O_10_S_2_Ni: C, 34.74; H, 4.01; N, 10.13; Found: C, 34.71; H, 4.06; N, 10.18.

### Refinement

C– and N-bound H atoms were placed geometrically [C—H = 0.93 and N—H = 0.86 Å] and refined using a riding model, with *U*_iso_(H)=1.2Ueq(N,*C*). Water H atoms were located on a difference map, and were refined isotropically with bond restraints O—H = 0.82 (2) Å and H···H = 1.35 (2) Å.

### Crystal data

**Table:**

[Ni(C_3_H_4_N_2_)_2_(H_2_O)_4_](C_10_H_6_O_6_S_2_)	*Z* = 1
*M**_r_* = 553.21	*F*_000_ = 286
Triclinic, *P*1	*D*_x_ = 1.656 Mg m^−^^3^
Hall symbol: -P 1	Mo *K*α radiation λ = 0.71073 Å
*a* = 8.285 (3) Å	Cell parameters from 2499 reflections
*b* = 8.925 (3) Å	θ = 2.5–27.5º
*c* = 9.088 (3) Å	µ = 1.12 mm^−^^1^
α = 107.705 (5)º	*T* = 273 (2) K
β = 101.628 (5)º	Plate, blue
γ = 111.967 (5)º	0.37 × 0.28 × 0.22 mm
*V* = 554.6 (3) Å^3^	

### Data collection

**Table:**

Bruker SMART 1K CCD diffractometer	2499 independent reflections
Radiation source: fine-focus sealed tube	2262 reflections with *I* > 2σ(*I*)
Monochromator: graphite	*R*_int_ = 0.011
*T* = 273(2) K	θ_max_ = 27.5º
φ and ω scans	θ_min_ = 2.5º
Absorption correction: multi-scan(SADABS; Bruker, 2001)	*h* = −10→10
*T*_min_ = 0.681, *T*_max_ = 0.790	*k* = −11→11
4834 measured reflections	*l* = −11→11

### Refinement

**Table:**

Refinement on *F*^2^	Secondary atom site location: difference Fourier map
Least-squares matrix: full	Hydrogen site location: inferred from neighbouring sites
*R*[*F*^2^ > 2σ(*F*^2^)] = 0.024	H atoms treated by a mixture of independent and constrained refinement
*wR*(*F*^2^) = 0.067	*w* = 1/[σ^2^(*F*_o_^2^) + (0.0346*P*)^2^ + 0.1772*P*] where *P* = (*F*_o_^2^ + 2*F*_c_^2^)/3
*S* = 1.08	(Δ/σ)_max_ = 0.001
2499 reflections	Δρ_max_ = 0.35 e Å^−^^3^
167 parameters	Δρ_min_ = −0.25 e Å^−^^3^
6 restraints	Extinction correction: none
Primary atom site location: structure-invariant direct methods	

### Special details

**Table:**

Geometry. All e.s.d.'s (except the e.s.d. in the dihedral angle between two l.s. planes) are estimated using the full covariance matrix. The cell e.s.d.'s are taken into account individually in the estimation of e.s.d.'s in distances, angles and torsion angles; correlations between e.s.d.'s in cell parameters are only used when they are defined by crystal symmetry. An approximate (isotropic) treatment of cell e.s.d.'s is used for estimating e.s.d.'s involving l.s. planes.
Refinement. Refinement of *F*^2^ against ALL reflections. The weighted *R*-factor *wR* and goodness of fit *S* are based on *F*^2^, conventional *R*-factors *R* are based on *F*, with *F* set to zero for negative *F*^2^. The threshold expression of *F*^2^ > σ(*F*^2^) is used only for calculating *R*-factors(gt) *etc*. and is not relevant to the choice of reflections for refinement. *R*-factors based on *F*^2^ are statistically about twice as large as those based on *F*, and *R*- factors based on ALL data will be even larger.

### Fractional atomic coordinates and isotropic or equivalent isotropic displacement parameters (Å^2^)

**Table:**

	*x*	*y*	*z*	*U*_iso_*/*U*_eq_	
Ni1	0.5000	0.5000	0.5000	0.02611 (9)	
N1	0.28615 (18)	0.55991 (17)	0.43827 (16)	0.0318 (3)	
O1W	0.31627 (16)	0.23254 (16)	0.43179 (17)	0.0400 (3)	
O2W	0.50259 (17)	0.42911 (17)	0.25854 (14)	0.0370 (3)	
S1	0.21612 (5)	0.14229 (5)	0.81936 (4)	0.02708 (10)	
C1	0.41626 (19)	0.11368 (19)	0.88348 (17)	0.0259 (3)	
C5	0.41915 (19)	0.01134 (18)	0.97747 (17)	0.0248 (3)	
O3	0.06491 (16)	−0.03557 (16)	0.71539 (15)	0.0406 (3)	
O2	0.18864 (17)	0.22940 (17)	0.96754 (15)	0.0407 (3)	
O1	0.25746 (18)	0.25029 (18)	0.72669 (17)	0.0436 (3)	
N2	0.1179 (2)	0.6991 (2)	0.4648 (2)	0.0478 (4)	
H7A	0.0826	0.7757	0.5081	0.057*	
C2	0.5648 (2)	0.1890 (2)	0.8392 (2)	0.0353 (3)	
H2A	0.5610	0.2560	0.7789	0.042*	
C3	0.7231 (2)	0.1653 (2)	0.8846 (2)	0.0390 (4)	
H3A	0.8235	0.2169	0.8541	0.047*	
C7	0.0278 (3)	0.5634 (3)	0.3116 (3)	0.0449 (4)	
H2B	−0.0831	0.5350	0.2335	0.054*	
C8	0.1323 (2)	0.4782 (2)	0.2957 (2)	0.0384 (4)	
H8A	0.1045	0.3792	0.2025	0.046*	
C6	0.2709 (3)	0.6926 (3)	0.5365 (2)	0.0459 (4)	
H6A	0.3560	0.7719	0.6425	0.055*	
C4	0.7313 (2)	0.0678 (2)	0.9726 (2)	0.0317 (3)	
H4A	0.8366	0.0526	1.0006	0.038*	
H2WA	0.570 (3)	0.510 (2)	0.241 (3)	0.057 (7)*	
H2WB	0.402 (3)	0.368 (3)	0.176 (3)	0.054 (6)*	
H1WA	0.215 (2)	0.179 (3)	0.357 (2)	0.062 (7)*	
H1WB	0.302 (3)	0.211 (4)	0.511 (2)	0.075 (9)*	

### Atomic displacement parameters (Å^2^)

**Table:**

	*U*^11^	*U*^22^	*U*^33^	*U*^12^	*U*^13^	*U*^23^
Ni1	0.02173 (14)	0.02598 (14)	0.02342 (14)	0.00944 (11)	0.00564 (10)	0.00513 (10)
N1	0.0287 (6)	0.0320 (7)	0.0311 (6)	0.0147 (5)	0.0101 (5)	0.0084 (5)
O1W	0.0273 (6)	0.0327 (6)	0.0433 (7)	0.0060 (5)	0.0060 (5)	0.0096 (5)
O2W	0.0350 (6)	0.0404 (7)	0.0274 (6)	0.0143 (5)	0.0094 (5)	0.0094 (5)
S1	0.02158 (17)	0.02921 (19)	0.02718 (19)	0.01217 (14)	0.00530 (14)	0.00956 (14)
C1	0.0207 (6)	0.0278 (7)	0.0256 (7)	0.0112 (5)	0.0059 (5)	0.0086 (5)
C5	0.0207 (6)	0.0249 (7)	0.0235 (6)	0.0089 (5)	0.0068 (5)	0.0063 (5)
O3	0.0256 (5)	0.0360 (6)	0.0415 (7)	0.0106 (5)	0.0003 (5)	0.0055 (5)
O2	0.0353 (6)	0.0498 (7)	0.0359 (6)	0.0267 (6)	0.0115 (5)	0.0088 (5)
O1	0.0405 (7)	0.0530 (7)	0.0522 (7)	0.0265 (6)	0.0175 (6)	0.0337 (6)
N2	0.0513 (9)	0.0465 (9)	0.0621 (10)	0.0348 (8)	0.0301 (8)	0.0216 (8)
C2	0.0299 (8)	0.0428 (9)	0.0413 (9)	0.0175 (7)	0.0154 (7)	0.0251 (7)
C3	0.0267 (8)	0.0513 (10)	0.0506 (10)	0.0182 (7)	0.0214 (7)	0.0308 (8)
C7	0.0343 (9)	0.0536 (11)	0.0540 (11)	0.0253 (8)	0.0156 (8)	0.0254 (9)
C8	0.0304 (8)	0.0403 (9)	0.0368 (9)	0.0181 (7)	0.0064 (7)	0.0083 (7)
C6	0.0470 (10)	0.0432 (10)	0.0400 (9)	0.0251 (8)	0.0129 (8)	0.0049 (8)
C4	0.0215 (7)	0.0382 (8)	0.0372 (8)	0.0145 (6)	0.0116 (6)	0.0168 (7)

### Geometric parameters (Å, °)

**Table:**

Ni1—N1^i^	2.0568 (14)	C1—C5	1.431 (2)
Ni1—N1	2.0568 (14)	C5—C4^ii^	1.422 (2)
Ni1—O2W^i^	2.0979 (13)	C5—C5^ii^	1.427 (3)
Ni1—O2W	2.0979 (13)	N2—C6	1.334 (2)
Ni1—O1W	2.0978 (13)	N2—C7	1.357 (3)
Ni1—O1W^i^	2.0978 (13)	N2—H7A	0.8600
N1—C6	1.315 (2)	C2—C3	1.407 (2)
N1—C8	1.378 (2)	C2—H2A	0.9300
O1W—H1WA	0.82 (2)	C3—C4	1.359 (2)
O1W—H1WB	0.82 (2)	C3—H3A	0.9300
O2W—H2WA	0.81 (2)	C7—C8	1.351 (2)
O2W—H2WB	0.81 (2)	C7—H2B	0.9300
S1—O2	1.4453 (12)	C8—H8A	0.9300
S1—O3	1.4508 (13)	C6—H6A	0.9300
S1—O1	1.4556 (13)	C4—C5^ii^	1.422 (2)
S1—C1	1.7811 (15)	C4—H4A	0.9300
C1—C2	1.369 (2)		
			
N1^i^—Ni1—N1	180.0	O1—S1—C1	106.33 (7)
N1^i^—Ni1—O2W^i^	91.34 (5)	C2—C1—C5	121.07 (13)
N1—Ni1—O2W^i^	88.66 (5)	C2—C1—S1	118.79 (12)
N1^i^—Ni1—O2W	88.66 (5)	C5—C1—S1	120.14 (10)
N1—Ni1—O2W	91.34 (5)	C4^ii^—C5—C5^ii^	119.05 (17)
O2W^i^—Ni1—O2W	180.0	C4^ii^—C5—C1	122.89 (13)
N1^i^—Ni1—O1W	87.22 (6)	C5^ii^—C5—C1	118.05 (16)
N1—Ni1—O1W	92.78 (6)	C6—N2—C7	107.87 (15)
O2W^i^—Ni1—O1W	91.48 (5)	C6—N2—H7A	126.1
O2W—Ni1—O1W	88.52 (5)	C7—N2—H7A	126.1
N1^i^—Ni1—O1W^i^	92.78 (6)	C1—C2—C3	120.14 (15)
N1—Ni1—O1W^i^	87.22 (6)	C1—C2—H2A	119.9
O2W^i^—Ni1—O1W^i^	88.52 (5)	C3—C2—H2A	119.9
O2W—Ni1—O1W^i^	91.48 (5)	C4—C3—C2	120.80 (14)
O1W—Ni1—O1W^i^	180.0	C4—C3—H3A	119.6
C6—N1—C8	104.96 (14)	C2—C3—H3A	119.6
C6—N1—Ni1	124.39 (12)	C8—C7—N2	105.88 (16)
C8—N1—Ni1	130.65 (11)	C8—C7—H2B	127.1
Ni1—O1W—H1WA	122.5 (16)	N2—C7—H2B	127.1
Ni1—O1W—H1WB	113.1 (19)	C7—C8—N1	109.81 (16)
H1WA—O1W—H1WB	107.7 (19)	C7—C8—H8A	125.1
Ni1—O2W—H2WA	115.4 (16)	N1—C8—H8A	125.1
Ni1—O2W—H2WB	121.1 (15)	N1—C6—N2	111.48 (16)
H2WA—O2W—H2WB	106.8 (18)	N1—C6—H6A	124.3
O2—S1—O3	113.08 (8)	N2—C6—H6A	124.3
O2—S1—O1	112.57 (8)	C3—C4—C5^ii^	120.88 (14)
O3—S1—O1	111.83 (8)	C3—C4—H4A	119.6
O2—S1—C1	106.98 (7)	C5^ii^—C4—H4A	119.6
O3—S1—C1	105.41 (7)		
			
O2W^i^—Ni1—N1—C6	37.27 (15)	S1—C1—C5—C4^ii^	−1.5 (2)
O2W—Ni1—N1—C6	−142.73 (15)	C2—C1—C5—C5^ii^	−0.5 (2)
O1W—Ni1—N1—C6	128.69 (15)	S1—C1—C5—C5^ii^	178.52 (13)
O1W^i^—Ni1—N1—C6	−51.31 (15)	C5—C1—C2—C3	0.6 (2)
O2W^i^—Ni1—N1—C8	−142.13 (15)	S1—C1—C2—C3	−178.51 (14)
O2W—Ni1—N1—C8	37.87 (15)	C1—C2—C3—C4	0.0 (3)
O1W—Ni1—N1—C8	−50.71 (15)	C6—N2—C7—C8	−0.1 (2)
O1W^i^—Ni1—N1—C8	129.29 (15)	N2—C7—C8—N1	0.2 (2)
O2—S1—C1—C2	−121.03 (14)	C6—N1—C8—C7	−0.1 (2)
O3—S1—C1—C2	118.35 (14)	Ni1—N1—C8—C7	179.38 (12)
O1—S1—C1—C2	−0.52 (15)	C8—N1—C6—N2	0.0 (2)
O2—S1—C1—C5	59.89 (13)	Ni1—N1—C6—N2	−179.51 (12)
O3—S1—C1—C5	−60.73 (13)	C7—N2—C6—N1	0.1 (2)
O1—S1—C1—C5	−179.60 (12)	C2—C3—C4—C5^ii^	−0.6 (3)
C2—C1—C5—C4^ii^	179.40 (15)		

Symmetry codes: (i) −*x*+1, −*y*+1, −*z*+1; (ii) −*x*+1, −*y*, −*z*+2.

### Hydrogen-bond geometry (Å, °)

**Table:**

*D*—H···*A*	*D*—H	H···*A*	*D*···*A*	*D*—H···*A*
O1W—H1WA···O3^iii^	0.82 (2)	2.022 (15)	2.7507 (18)	148 (2)
O1W—H1WB···O1	0.82 (2)	2.018 (14)	2.788 (2)	156 (3)
O2W—H2WA···O1^i^	0.81 (2)	1.962 (10)	2.7496 (19)	161 (2)
O2W—H2WB···O2^iv^	0.81 (2)	1.96 (2)	2.7979 (18)	173 (2)
N2—H7A···O3^v^	0.86	2.19	2.981 (2)	154

Symmetry codes: (iii) −*x*, −*y*, −*z*+1; (i) −*x*+1, −*y*+1, −*z*+1; (iv) *x*, *y*, *z*−1; (v) *x*, *y*+1, *z*.
